# EEG spectral power during REM sleep in patients with frontal brain tumor

**DOI:** 10.1186/s12883-023-03243-1

**Published:** 2023-05-19

**Authors:** Laura Victoria Ortega-Leonard, Yolanda del Río-Portilla

**Affiliations:** 1 Laboratorio de Sueño, Facultad de Psicología, Universidad Nacional Autónoma de México (UNAM), Av. Universidad 3004, Ciudad de Mexico, C. P. 04510 México; 2 Coordinación de Psicobiología Y Neurociencias, Facultad de Psicología, UNAM, Ciudad de México, México

**Keywords:** REM sleep, EEG spectral power, Frontal lobe, Brain tumors

## Abstract

**Background:**

The main objective of this research was to analyze the characteristics of electrical activity in the brain during REM (Rapid Eye Movements) sleep, by using an experimental model a pathology that affects the frontal lobes, such as brain tumors. In addition to determining the impact of variables such as the frontal area (dorsolateral, medial and orbital), laterality and size of the lesion; as well as the demographic and clinical characteristics of the patients evaluated.

**Methods:**

By using polysomnographic recordings, 10 patients were evaluated. We obtained power spectra through a homemade program. For quantitative EEG (Electroencephalogram) (qEEG) analysis, the Fast Fourier Transform (FFT) algorithm was used to obtain the spectral power of each participant, channel, and frequency band.

**Results:**

Sleep architecture and spectral power was found to be modified in patients compared to normative values. Other sociodemographic and clinical characteristics of the patients were also influenced, such as age range and antiepileptic drugs.

**Conclusions:**

Brain tumors in the frontal lobe can modify the rhythmogenesis of REM sleep, possibly due to changes of brain plasticity as an effect of the pathology. In addition to this, through this study we were able to show the association between neuroanatomical and functional changes, on the characteristics of brain electrical activity in patients with frontal brain tumor. Finally, this qEEG analysis technique allows, on the one hand, to deepen the knowledge and relationship between psychophysiological processes and, on the other hand, to be able to guide therapeutic decisions.

## Background

REM (Rapid Eye Movements) sleep is characterized by the activation of the Electroencephalogram (EEG), by a low-voltage mixed frequency electroencephalographic activity, in addition to muscle atony and episodic bursts of REMs [1, 2]. REM sleep is regulated by various brain structures, including the frontal lobes, which are known to play an important role during REM sleep in the regulation of eye movements [3–5]. According to the descriptions of the distribution of brain activity by Positron Emission Tomography (PET) [6, 7], there is a decrease in the metabolic rate in the prefrontal cortex during REM sleep. This corroborates what has been previously reported about the relationship between changes in activity in this area and the characteristics of dreams.

In terms of brain electrical activity through quantitative EEG (qEEG) analysis; REM sleep has been mainly associated with rapid oscillations in the frontal regions. For example, a higher spectral power during REM sleep compared to nonrapid eye movement (NREM) sleep and wakefulness is shown in the Gamma frequency [8–10]. Regarding laterality, there are few studies that have researched this aspect during REM sleep; for instance, in young healthy adults, a right frontal lateralization in Theta and Beta bands was reported to having been found, which could be explained by cognitive aspects occurred during this stage of sleep [11].

On the other hand, the electrophysiological patterns of sleep can be modified due to a brain pathology, which allows us to know in depth what happens when these lesions affect a particular area of the brain. In this regard, it has been found that in patients with brain tumor within frontal regions show changes in brain electrical activity: the presence of a spectral power peak in the Alpha rhythm range is observed during REM sleep, being greater in the contralateral hemisphere affected by tumor; which in turn may indicate a focus of activation and fragmentation of sleep that is formed in the presence of brain tumors [12]. However, spectral power analysis was carried out only in the first three sleep cycles, which leaves aside the longest periods of REM sleep, which predominate in the second half of the night. Likewise, the range of frequencies up to 24 Hz was analyzed, ignoring the fast frequencies above this limit, such as the high segment of Beta band and Gamma band, which have been reported mainly present in this stage of sleep [8–10].

It is worth mentioning that the frontal lobes are further divided into regions of the Dorsolateral Prefrontal Cortex (dlPFC), Medial Prefrontal Cortex (mPFC), and Orbital Prefrontal Cortex (oPFC). In a previous study, changes were found in the activation of these regions (dlPFC and mPFC) in the Beta and Theta bands in epileptic patients; however, the neuroanatomical involvement occurred in different brain regions, mainly in temporal lobe [13].

The frontal lobes have previously been associated with different aspects of REM sleep, such as the regulation of eye movements and cognitive changes during dreams. This is related to the cortical oscillations measured by the spectral power densities of EEG; however, such brain electrical activity in frontal lobes during REM sleep remains poorly understood. Studies are therefore required that involve these appropriately delimited lobes as well as an adequate cerebral electric activity study by analysis of spectral power of EEG and incorporation of rapid bands like Gamma.

Therefore, the aim of the current investigation was to analyze brain electrical activity (spectral power) during REM sleep in patients with frontal brain pathology. Likewise, to evaluate if there are differences according to the division of the frontal area (dorsolateral, medial and orbital), laterality, size of the lesion or with posterior areas of the cortex; as well as in demographic and clinical characteristics of the patients evaluated.

## Methods

### Participants

The sample consisted of ten people with diagnoses of glioma-type tumor in the frontal lobe, with mean age of 43.4 years (Standard deviation = 11, range = 26–57, 3 women), all of whom attended to the National Cancer Institute in Mexico City. Inclusion criteria included the absence of treatment (surgery, chemotherapy or radiotherapy) and absence of neurological, psychiatric or autoimmune comorbidity. Exclusion criteria included pre-existing neurological disorder or a brain injury and substance abuse disorder. As well as large lesions in brain regions beyond the areas of interest (or displacement of the tumor such that over one-third of its total volume invaded other cerebral regions).

Participants were required not to have any ingestion of caffeine or other stimulating substances for at least two days before the PSG record. Use of medications known to cause effects on sleep were controlled, however, some patients were under treatment with antiepileptics to control seizures caused by the tumor (two with Carbamazepine, three with Levetiracetam and two patients with two antiepileptics at the same time Valproato-Levetiracetam and Valproato-Phenytoin). All the patients were right handed and were registered before tumor resection.

To determine the diagnosis, location (medial, orbital or dorsolateral), laterality and for tumor and edema measurement, brain images were analyzed by structural Magnetic Resonance Imaging (MRI) using 8-channel Magnetic Resonance General Electric Signa Excite II, Tesla 3 equipment. Sequence T1 and T2 MRI were utilized. For the anatomical mapping of lesions, the study had the support of expert neuroradiologists who were blind to our objectives.

In order for the participant to be included, the lesion had to be localized in frontal area. Following the anatomical mapping procedure, patients were divided into three groups [14] according to the precise site and extent of their lesions: 1) the mPFC group, where damage involved the area between the superior frontal sulcus and medial orbitofrontal gyrus (Brodmann areas 8,9,10,11,12,24,32); 2) the oPFC group, when the tumor was located between the medial sulcus of the H-shaped gyrus and the lateral surface of the third frontal convolution (Brodmann areas 10,11,13,47); and 3) the dlPFC group, where the tumors affected the region from the superior frontal sulcus to the inferior frontal sulcus (Brodmann areas 8,9,10,11,44,45,46,47). Additionally, a grouping was carried out according to the size of the volume and edema of the tumors in small (< 10cm^3^), medium (between 10.001 and 50cm^3^) and large (> 50cm^3^).

### Procedure

This study was approved by the ethic committees of the Master's and PhD Program in Psychology of the UNAM and the National Cancer Institute in Mexico (018/001/OMI) (CEI/1219/17). All participants signed the informed consent form. The research complied with the principles of the Declaration of Helsinki for human study. All participants gave their consent to participate in our research.

Polysomnography recording was performed at the Sleep Laboratory of the Faculty of Psychology at the UNAM or in the home of patient, taking care that there were favorable noise and climate conditions for the studies. A statistical analysis of brain electrical activity was carried out between the patients who carried out their sleep recording at home and those who carried it out in the laboratory, without finding significant differences.

Patients spent two nights at the laboratory, the first one for adaptation to recording procedures. Electrical activity during spontaneous sleep from the second night spent at the laboratory was analyzed in nine patients and the first and only night of a patient. It was decided that this patient should be part of the sample, since by means of a previous statistical analysis of the PSG values between night one or habituation and night two, it was found that only the sleep onset latency showed significant differences (*p* < 0.05), however, comparing this values of the latency of one night with the expected parameters [15], they do not differ significantly (*p* ≥ 0.05).

For the recording of brain electrical activity, 19 electrodes were placed according to 10/20 international system, with monopolar montage from Fp1, Fp2, F3, F4, C3, C4, T3, T4, P3, P4, O1, and O2 referred to contralateral earlobes (A1 and A2), sampled at 400 Hz and filters set at 0.5 and 70 Hz. In addition to right and left electrooculograms and submental electromyograms were also applied to measure eye movements and muscle tone, respectively, and were placed according to the recommendations of the American Academy of Sleep Medicine (AASM) Version 2.5 [16]. For the recording and amplification of the signal, a Cadwell polygraph, with Easy EEG program version 2.1 was used. The sleep recording started from the usual sleep time of participant, and they were also allowed to sleep until spontaneous awakening the next day or until they had completed their usual hours of sleep determined by a sleep diary.

### Signal analysis

Physiologic signals were digitized for off-line analysis of spontaneous sleep for the second (nine patients) or one night (one patient) of recording and prior wakefulness with eyes closed. Sleep-stages were identified computer recordings on 30-s epochs according to the criteria of the AASM Version 2.5 [16], by two sleep experts trained in sleep staging. Subsequently, the sleep hypnograms were graphed and the PSG parameters were compared with the normative parameters of Hertenstein and cols. [15], which provides reliable reference data for the characterization of polysomnographic variables and spectral power in the stages of stages.

Standard indicators for identified REM sleep were desynchronized EEG, minimization of muscle tone and the emergence of REMs. The first or second REM sleep stages from the second half of the night were selected, inspected for artifacts, and analyzed, because REM sleep episodes are longer compared to the first half of the night, in addition, because to the total number of REM sleep cycles were variable among patients.

For the analyses quantitative EEG (qEEG), Fast Fourier Transform (FFT) algorithm was used to obtain the power spectral, which gives information on the absolute power or intensity of the output of the generators of a particular oscillatory activity [17].

Spectral power was average over 1 Hz; it was obtained for each participant, channel, and the following frequency bands: Delta (1–3.5 Hz), Theta (3.5–8 Hz), Alfa (8–12 Hz), Sigma (12–14 Hz), Beta1 (16–24 Hz), Beta2 (24–32 Hz) and Gamma (32–48 Hz), through the POTENCOR program, in which each 1-s epoch was carefully inspected for artifacts, and then all artifact-free, 1-s epochs were FFT (1–50 Hz). This program initially calculates the FFT and then determines the cross-correlation spectrum in the time domain by means of Pearson product-moment coefficients using voltage values for each channel after filtering the signals in the frequency domain [17].

### Statistical analyses

Statistical analyses were carried out with the software IBM SPSS Statistics 21 (Statistical Package for the Social Sciences [SPSS] Inc., Chicago, IL, USA). The level of statistical significance was set at *p* = 0.05 for all analyses. The Mann–Whitney U test was performed to evaluate differences between PSG variables of sleep architecture of patients with brain tumors and normative values. Spectral power values were log transformed before to any statistical test to approximate them toward normal distribution. The spectral power values were compared between patients with brain tumors and normative values [15]. To determine if there are differences by sex, laterality of the tumor and antiepileptic treatment in the spectral power was used the Mann–Whitney U test. An analysis was carried out using the Kruskal–Wallis Rank test, to know if there were differences between the types of antiepileptic drugs and the size of the lesion (small, medium, and large).

## Results

### Sleep architecture

Sleep architecture data indicate changes in the sleep patterns of frontal brain tumor patients. They have a shorter sleep onset latency (*p* = 0.0) and percentage of REM sleep (*p* = 0.02); as well as a longer wake time after sleep onset (*p* = 0.02) and percentage of stage 2 (*p* = 0.0). Despite this, sleep efficiency was within normal range [15] (Table 1).

**Table 1:** Sleep parameters and results of the comparison analyzes with the criteria values

Sleep Parameters	Mean	Standard Deviation (SD)	Comparison criteria values *p*
Time from sleep onset until final awakening (TST) (min)	324	64.2	0.09
Sleep Onset Latency (SOL) (min)	6	2.6	0.0**
REM sleep latency (min)	76	31.1	0.68
Wake After Sleep Onset (WASO) (min)	95	45	0.02*
% Stage 1 Sleep	8	6.3	0.28
% Stage 2 Sleep	64	7.2	0.0*
% Stage 3 Sleep	10	6.7	0.53
% Stage REM Sleep	16	5	0.02*
% Sleep Efficiency (SE)	76	10.6	0.11

In bold are the values that presented significant differences between in the variables of sleep architecture between patients with tumor and the normative parameters of Hertenstein and cols. [15]

^*^p < 0.05, **p < 0.01

### REM sleep EEG activity for different bands

**Fig. 1:**
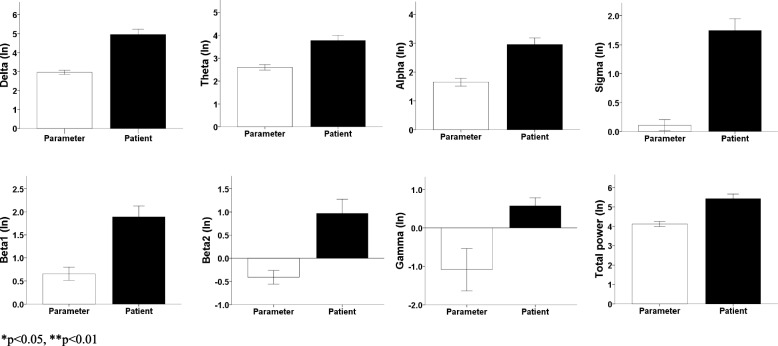
Spectral power values by frequency band. Average values of spectral power (presented in logarithms [In]) for each band of the patients with frontal brain tumor in comparison with the parameters [15]

Figure 1 shows the average spectral power for REM sleep, for all electrodes sites and for each of the bands. Where higher spectral power can be observed by patients compared to the normative values [15].

### REM sleep EEG activity for location of the frontal area and laterality

According to the location by areas of the frontal lobe, in six patients the tumor was in the dorsolateral area, in four in the medial area and none in the orbital area. Regarding laterality, in six patients the tumor was in the right hemisphere and four in the left hemisphere. Mann–Whitney U test results indicated no significant main effect for the location of the frontal area and the laterality (*p* ≥ 0.05).

### REM sleep EEG activity for size of the lesion

The volume and edema values were combined to calculate the total size of the lesion, whose average value was 71.7 cm^3^, in a range of 7.6 to 226.2 cm^3^. Additionally, a grouping was made according to the size of the lesion in small (< 10cm^3^), medium (between 10.001 and 50cm^3^) and large (> 50cm^3^). No significant differences among sizes of the lesion were found (*p* ≥ 0.05).

### REM sleep EEG activity for sex, age range and antiepileptic treatment

No significant differences were found in the group of patients by sex. On the contrary, the age range showed significant differences, specifically, patients in the range of 41 to 50 years, obtained greater power in the delta 2 band (*p* = 0.03), Gamma (*p* = 0.03) and the total power (*p* = 0.04). Likewise, differences were observed in antiepileptic treatment, since there was a decrease in the theta band in patients taking medication (*p* = 0.03).

### REM sleep EEG activity for anterior vs posterior electrodes

**Fig. 2:**
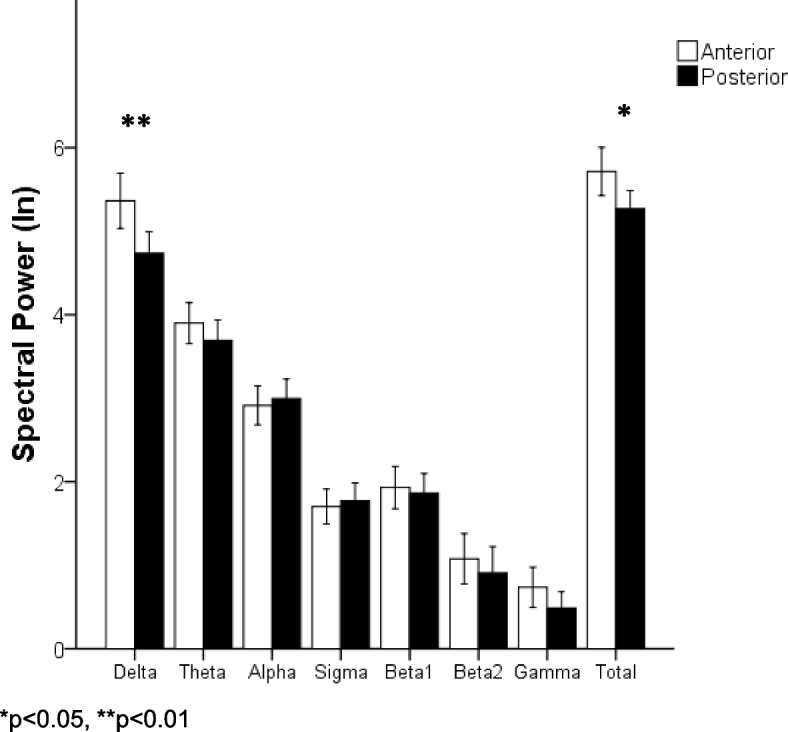
Spectral power between anterior and posterior electrodes. Average values of spectral power of the anterior and posterior electrodes for each band of the patients with frontal tumor (presented in logarithms [In]). The white bars belong to the anterior electrodes, while the black bars belong to the posterior electrodes

Comparisons between anterior vs posterior electrodes revealed significant difference for the Delta (*p* = 0.003) and total (*p* = 0.02) bands. In which the anterior electrodes have greater power compared to the posterior electrodes (Fig. 2).

## Discussion and conclusion

Regarding results of sleep architecture, our findings aligned well with previous findings in patients with brain pathology [18–20], showing sleep deficiencies characterized by the presence of high levels of sleep fragmentation, the decrease in sleep-onset latency (reported as an assessment of sleep debt [1] and irregularities in the phase percentage (stage 2 and REM sleep).

As regards to the spectral power analysis, differences were revealed in all the bands between the patients and criterion values, which indicates that these lobes also have a participation in the power distribution mechanism of an EEG signal. In addition to this, it can also be considered as an effect of plasticity or compensatory reaction in the sample recorded in this study.

Likewise, to know if this participation of the frontal lobes in the spectral power differs according to the area of the frontal lobe affected by the tumor, a statistical analysis was carried out that did not show significant differences. Although, previously, differences in the activation of the frontal areas during REM sleep have been reported in people without pathology and given by other methods of study of brain activity such as PET [6, 7], where less activation was observed in dorsolateral prefrontal regions and lateral orbital, as well as greater in medial prefrontal regions; however, these differences were not observed in brain electrical activity in the patients registered in our study.

According to the laterality of the tumor, no significant differences were found either, which contrasts with what was previously observed in young healthy adults of a right frontal lateralization in Theta and Beta bands [11]. The fact that no changes in laterality were observed in the patients registered in this sample could indicate an impact of the tumor in this aspect; however, these findings must be taken with caution, given the discrepancy in the age range of both studies.

With respect to laterality of cerebral electrical activity in the presence of frontal lobe pathology, in a previous study in patients with tumor, an effect of the hemispheric laterality of the tumor was observed in the Alpha rhythm range [12]. Despite the similarities in the type of sample between this study and ours, there are some discrepancies in the normative values used for the comparison of the spectral power data and in the analysis of the sleep cycles used, which could explain the divergences between the results in laterality. Finally, regarding the characteristics of the tumors, the size of these did not show an effect on the spectral power, despite the variability in their size.

In the sociodemographic characteristics, the patients presented significant differences according to the age range of 41 to 50 years, with greater power in the Delta and Gamma bands. The increase in Gamma could be partly explained by the observed increase in rapid activity with age [15], while the increase in Delta could be a repercussion of the presence of the tumor [21, 22]. On the other hand, the lower Theta power observed in those patients taking antiepileptic drugs could represent an effect of these drugs on sleep [23, 24].

In the comparison between the anterior and posterior electrodes, to find out if there were differences between anterior–posterior regions, it was possible to observe a greater power in the Delta band in anterior regions, which may be due to what was previously reported by Bernardi and cols.[25], regarding the so-called frontal-central waves or sawtooth waves characteristic of REM sleep, which are within the range of the Delta band (2.5–3.0 Hz) and a maximum power between the vertex electrodes and frontal. The increase in Delta could be a repercussion of the presence of the tumor, since greater activity in this band has been previously reported in patients with brain tumors [21, 22].

Given the findings reported in this study, it can be observed that an alteration in the frontal lobe, using an experimental model as a pathology that affects this area, can generate changes in the rhythmogenesis of REM sleep, observed in terms of spectral power through the quantitative EEG analysis. The same can be observed in the face of a low amount of REM sleep in the face of a deprivation of this stage of sleep and in which the frontopolar regions are affected. This suggests that the plastic reorganization and the flow of information to other brain regions are altered and, therefore, the processing of tasks that require the participation of the supervisory control of the prefrontal regions [26]. Likewise, within the clinical field, changes in spectral power could explain the changes observed in sleep architecture, such as the increase in the number of awakenings or sleep fragmentation, due to pathological cortical activation [27] or an activation focus mainly related to a power peak in the Alpha band [12].

Our findings provide neurophysiological evidence that power spectral analysis, using the EEG technique; It is a useful tool and an important complement to other neuroimaging techniques to investigate changes in brain activity during sleep using objective parameters.

It is important to mention that although there was not a large sample of patients evaluated, which may be a limitation of our research, we tried to privilege a greater control of the variables studied in these patients, among which stand out; the type of pathology and the neuroanatomical delimitation of the lesion. Also, normality criteria of the spectral power values valid for the evaluated population were used. Another important aspect to consider is the treatment with anticonvulsants by patients. Although the statistical analysis showed significant differences in spectral power only in the Theta band, further investigation is recommended in this regard. Finally, this study allows us, on the one hand, to gain an in-depth understanding of what happens when this type of injury affects a certain area of the brain and, on the other hand, it also allows us to observe the changes in plasticity that lead to a reorganization of brain function during this state.

## Data Availability

The datasets used and/or analysed during the current study are available from the corresponding author on reasonable request.

## Abbreviations

REM: Rapid Eye Movements
PSG: Polysomnographic
EEG: Electroencephalogram
qEEG: Quantitative Electroencephalogram
FFT: Fast Fourier Transformation
PET: Positron Emission Tomography
NREM: Nonrapid eye movement
dlPFC: Dorsolateral Prefrontal Cortex
mPFC: Medial Prefrontal Cortex
oPFC: Orbital Prefrontal Cortex
MRI: Magnetic Resonance Imaging
SPSS: Statistical Package for the Social Sciences
AASM: American Academy of Sleep Medicine
